# Carbon, cesium and iodine isotopes in Japanese cedar leaves from Iwaki, Fukushima

**DOI:** 10.1007/s10967-016-4830-5

**Published:** 2016-04-21

**Authors:** Sheng Xu, Gordon T. Cook, Alan J. Cresswell, Elaine Dunbar, Stewart P. H. T. Freeman, Xiaolin Hou, Helen Kinch, Philip Naysmith, David W. C. Sanderson, Luyuan Zhang

**Affiliations:** 1 Scottish Universities Environmental Research Centre, East Kilbride, G75 0QF UK; 2 Center for Nuclear Technologies, Technical University of Denmark, 4000 Roskilde, Denmark; 3 Fukushima University, Fukushima, 960-1296 Japan; 4 Xi’an AMS Center, SKLLQG, Institute of Earth Environment, CAS, Xi’an, 710061 China

**Keywords:** Japanese cedar leaf, ^14^C, ^129^I, ^134,137^Cs, Fukushima nuclear accident

## Abstract

Japanese cedar leaves from Iwaki, Fukushima were analyzed for carbon, cesium and iodine isotopic compositions before and after the 2011 nuclear accident. The Δ^14^C values reflect ambient atmospheric ^14^C concentrations during the year the leaves were sampled/defoliated, and also previous year(s). The elevated ^129^I and ^134,137^Cs concentrations are attributed to direct exposure to the radioactive fallout for the pre-fallout-expended leaves and to internal translocation from older parts of the tree for post-fallout-expended leaves. ^134^Cs/^137^Cs and ^129^I/^137^Cs activity ratios suggest insignificant isotopic and elemental fractionation during translocation. However, fractionation between radioiodine and radiocesium is significant during transportation from the source.

## Introduction

Significant radionuclide activities were released into the environment as a consequence of the serious damage to the Fukushima Dai-ichi Nuclear Power Plant (FDNPP) following the earthquake and tsunami that occurred on 11th March 2011. This resulted in a large area in the Fukushima region being highly contaminated due to deposition of radioactive debris. In particular, as a large proportion of the land in Fukushima is covered by forest, it is important to understand the levels and behaviors of the deposited radionuclides in forest systems in support of post-accident decontamination procedures. Accordingly, numerous data on Fukushima-derived radionuclides in the forest environment of Fukushima have been reported recently [1–9]. These studies mainly focused on investigations of local distributions, post-accident uptake and translocation of radiocesium in different forest systems. However, the activities and behaviors of other long-lived radionuclides (i.e. ^129^I and ^14^C with half-lives of 1.57 × 10^7^ and 5730 years, respectively) in these systems have been sparsely addressed. In particular, ^14^C is an important radionuclide for regional radiological assessment because it gives a significant fraction of the effective dose to the general public via the atmosphere-agricultural food-ingestion pathway. However, ^14^C remains one of Fukushima’s most understudied radionuclides [10]. In addition to natural cosmogenic sources, anthropogenic ^14^C can be produced by the nuclear reactions ^14^N(n,p)^14^C, ^17^O(n,α)^14^C and ^13^C(n,γ)^14^C in nuclear reactors and nuclear weapons testing. A total of 213 PBq ^14^C from atmospheric nuclear weapons testing and 44 TBq from the Chernobyl accident in 1986 were estimated to have been released into the environment [11, 12]. In our previous study of ^14^C in annual rings of a 30-years-old Japanese cedar from Iwaki [13], local fossil fuel combustion resulted in a reduction in the Δ^14^C values compared to the accepted clean-air environment. The source was identified as originating from increasing traffic on two nearby expressways in the 1990’s. Meanwhile, a small but visible ^14^C pulse observed in the 2011 tree ring in Iwaki was thought to imply release from the Fukushima accident. Within the context of this background, the current work further investigates the radioactivity levels of long-lived radionuclides, ^14^C and ^129^I as well as ^134,137^Cs, in the leaves of Japanese cedar (*Cryptomeria japonica*) that were collected from the same site as the tree ring samples in [13]. To our best knowledge this is the first report of ^14^C and ^129^I analyses in the post-accident Japanese cedar forest system, which is widely distributed in the Fukushima region.

## Experimental

**Fig. 1:**
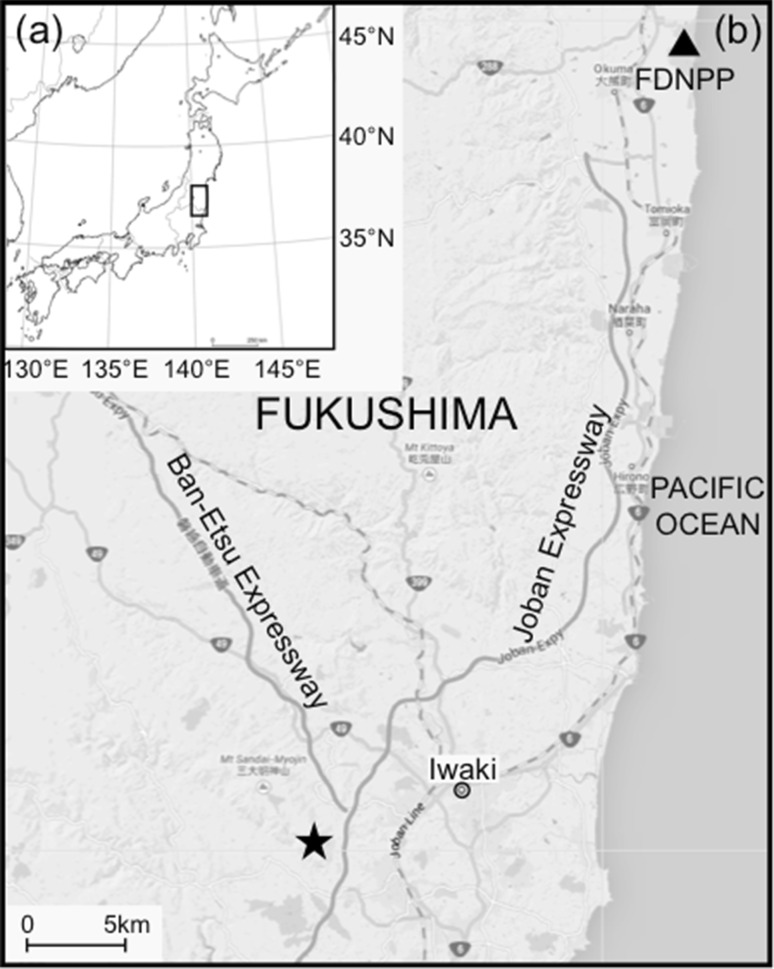
**a** Japanese map. The rectangle stands for the studying area. **b** Enlarged map of the rectangle in (**a**) showing sampling site marked with a star

The sampling site is located at Iwaki, Fukushima (37°0.53′N and 140°48.57′E), ~50 km southwest of the FDNPP (Fig. 1). Two branches (living and very recently dead) of the cedar were collected in January 2013. It is difficult and sometimes impossible to identify when a cedar branch fell from a tree. However, as the leaves on the fallen branch remained quite green in colour, this strongly suggests that a short time had elapsed since it fell. Thus, it is considered highly likely that the leaves sprouted before the Fukushima accident and the branch fell during the period of 2011 or 2012. Here we treat them as the pre-fallout-expended leaves (sprouted or developed before the 2011 accident) following the term used in [4]. In contrast, the living leaves, collected from the most recent growth, are catalogued as the post-fallout-expended leaves as they sprouted (or developed) in spring-summer 2012.

For ^14^C determination, several pieces of leaf were taken from the top, middle and base of the branches. They were then chemically treated by the routine acid–base–acid (A–B–A) procedure. The treated samples were combusted at 850 °C to obtain CO_2_. The purified CO_2_ samples were reduced to graphite, and the ^14^C/^13^C ratios in the prepared graphite samples were measured using the 5 MV accelerator mass spectrometer (AMS) at the Scottish Universities Environmental Research Centre (SUERC). Aliquots of CO_2_ were measured for δ^13^C by conventional isotope ratio mass spectrometry (IRMS) using a VG SIRA 11. A detailed description of the chemical pretreatment and graphitisation, the AMS procedures and data reduction are presented elsewhere [13, 14].

Iodine in the leaf samples was extracted by combustion followed by trapping with a 0.4 M NaOH–0.05 M NaHSO_3_ solution [15]. An aliquot of the trapping solution was taken for ^127^I determination by inductively coupled plasma mass spectrometry (ICP-MS) at the Technical University of Denmark. The iodine in the remaining trapping solution was further separated using solvent extraction after the addition of 2 mg of ^127^I carrier and the separated iodine, in the form of iodide, was precipitated as AgI for ^129^I/^127^I ratio measurement conducted by AMS at SUERC [16].

A gamma spectrometer with a coaxial Ge detector (EG&G ORTEC LoAx-51370/20P) was used for ^134^Cs and ^137^Cs measurement at SUERC. Details of the measurement procedure and data reduction have been reported elsewhere [17].

## Results and discussion

Table 1 lists the analytical results for δ^13^C and Δ^14^C in the leaf samples. The δ^13^C results are expressed as per mil deviations from the Vienna Pee Dee Belemnite (VPDB) standard. The ^14^C data are reported as Δ^14^C i.e. per mil deviations from the primary standard [0.7459 times the activity of NBS oxalic acid II (SRM-4990C)]. Table 2 lists the analytical results for ^134,137^Cs and ^127,129^I concentrations in this study and some previous literature values. All measured and cited ^134,137^Cs and ^131^I activities have been decay-corrected to 11th March 2011 when the FDNPP was shut down.

**Table 1:** δ^13^C and ^14^C results of Japanese cedar leaves from Iwaki, Fukushima

Material	Lab code	Sampling part	Possible sprouted year	δ^13^C (‰)	Δ^14^C (‰)
Fallen leaf	SUERC-46608	Base	Before 2011	−30.0	48.9 ± 0.1
	SUERC-46609	Middle	Before 2011	−30.5	51.0 ± 0.2
	SUERC-46610	Top	Before 2011	−30.0	45.8 ± 0.1
	Weighted mean			−30.2	47.8 ± 1.3
Living leaf	SUERC-46611	Base	After 2011	−29.1	32.1 ± 0.1
	SUERC-46612	Middle	After 2011	−30.0	34.1 ± 0.1
	SUERC-46613	Top	After 2011	−30.0	–
	Weighted mean			−29.7	33.1 ± 1.0

**Table 2:** Cesium and iodine isotopic compositions of Japanese cedar leaves from the Iwaki area, Fukushima*

Materials	Sampling date	^134^Cs (kBq kg^−1^)	^137^Cs (kBq kg^−1^)	^127^I (μg g^−1^)	^129^I (10^9^ atom g^−1^)	^131^I (kBq kg^−1^)	^134^Cs/^137^Cs (Bq Bq^−1^)	^129^I/^127^I (10^−7^ atom atom^−1)^	^129^I/^137^Cs (10^−6^ Bq Bq^−1^)
This study									
Fallen leaves	14 Jan 2013	3.15 ± 0.27	3.18 ± 0.05	3.06 ± 0.01	3.57 ± 0.03		0.99 ± 0.08	2.46 ± 0.03	1.57 ± 0.03
Living leaves	14 Jan 2013	1.29 ± 0.03	1.32 ± 0.04	0.390 ± 0.001	1.56 ± 0.02		0.98 ± 0.04	8.38 ± 0.11	1.66 ± 0.05
Hosoda et al. [23]									
Living leaves	18 Mar 2011	5.09 ± 0.08	5.19 ± 0.12			516.3 ± 1.4	0.98		2.22 ± 0.05
Living leaves	18 Mar 2011	5.62 ± 0.08	5.71 ± 0.12			349.2 ± 1.1	0.98		1.36 ± 0.03
Soil	18 Mar 2011	0.27 ± 0.01	0.30 ± 0.02			22.6 ± 0.2	0.89		1.68 ± 0.11
Soil	18 Mar 2011	0.35 ± 0.01	0.32 ± 0.03			22.2 ± 0.2	1.1		1.55 ± 0.15
Furuta [24]									
Living leaves	29 Mar 2011		4.5			780.8			3.87
Fallen leaves	29 Mar 2011		29			2602.8			2.00

* ^134,137^Cs and ^131^I decay-corrected to 14:46 of the 11th March 2011 when the reactors were shut down

## ^14^C activities

The Δ^14^C values from three different parts of the defoliated sample ranged from 45.8 to 51.0 ‰ with a weighted mean and one standard deviation of 47.8 ± 1.3 ‰, whereas those of the living sample varied from 32.1 to 34.1 ‰ with a weighted mean value of 33.1 ± 1.0 ‰. Obviously, the samples of living leaves have about 15 % less ^14^C than the defoliated leaves.

The ^14^C fixed in a tree by photosynthesis is considered to be a proxy for the ^14^C concentration in the ambient atmosphere during the growth period of the leaves. Among the chemical fractions within the tree, the alpha cellulose of the tree ring is considered the most reliable for determining the ^14^C incorporated at the time of growth [18]. Plant leaves are often useful for investigating ^14^C concentrations in the air during the timespan of leaf growth [19], however, in the case of cedar leaves, ^14^C values need to be integrated over a lifespan of several years. Sakurai et al. [20] showed that the ^14^C concentrations in leaves reflect the ambient atmospheric ^14^C concentration during the year that the leaves were sampled, or the year prior. In their study, the ^14^C concentrations in 3 pine needles were higher than those in paired bamboo leaves by 0.2–0.4 pMC. This was considered to be an indication of the longer lifespan of pine needles compared to that of bamboo leaves.

**Fig. 2:**
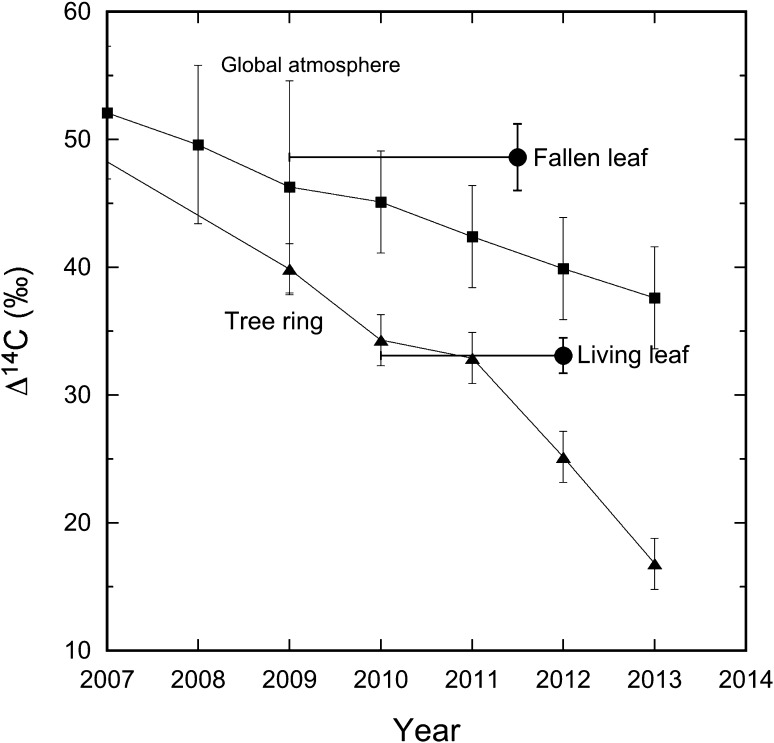
Temporal variations of Δ^14^C in Iwaki Japanese cedar leaves (this study) and tree rings [13] in comparison with global atmospheric ^14^C [21]

Figure 2 compares the Δ^14^C values in leaf samples with those in the global atmosphere represented by a monthly mean of May–August measurements of atmospheric CO_2_ and tree rings from Northern Hemisphere Zone 2 [21], where May–August represents the approximate growth period of these trees in the Fukushima area. The leaf values are also compared with values for rings from the same tree. The Δ^14^C values in the post-fallout-expended leaves are lower than the global atmospheric level in the sampling year of 2012 but higher than that in the contemporary tree ring of year 2012 (26.7 ± 1.9 ‰ in early wood and 23.6 ± 2.1 ‰ in late wood) [13]. However, they are comparable with values for the 2010 and 2011 rings [13]. Thus, the relatively higher Δ^14^C values in 2012 leaves compared to the contemporary tree ring might suggest that the leaves fixed ^14^C from previous years (i.e. 2011 and even early years) in addition to the current year of 2012. Similar results have been observed in pine needles and bamboo leaves in Yamagata in 2011–2012 [20].

The Δ^14^C values in the pre-fallout-expended leaves were higher than those in the contemporary global atmosphere. Normally, this might be attributed generally to discharges or releases from the operating reactors or a nuclear accident, as observed in many nuclear sites around the world [11]. However, it might not be the case in this study for the following reasons. As described above, the fallen leaves were most likely defoliated in 2011 or 2012. However, their Δ^14^C values are significantly higher than those in the tree rings of 2011 or 2012. Instead, they are comparable with those in the pre-2009 rings, within the error margin of the analytical uncertainty (Fig. 2). This fact is compatible with a lifespan of several years for the evergreen’s leaf. Hence, the higher Δ^14^C values in the pre-fallout-expended leaves, with respect to the contemporary rings of years 2011 or 2012, would suggest that the ^14^C in the leaves derived largely from the high ^14^C values in years prior to 2009, which reflects a potential lifespan of 3–4 years for the Japanese cedar leaf.

In summary, our present results fully support that the evergreen leaves may contain ^14^C fixed from previous years, as observed previously [20, 22]. As a result, care should be taken when ^14^C in the leaf of an evergreen tree is considered as a proxy for atmospheric ^14^C with an annual time resolution.

### ^134,137^Cs activity concentrations and ^134^Cs/^137^Cs activity ratio

^134^Cs and ^137^Cs concentrations in the pre-fallout-expended leaves are 3.15 ± 0.27 kBq kg^−1^ and 3.18 ± 0.05 kBq kg^−1^, respectively, resulting in a ^134^Cs/^137^Cs activity ratio of 0.99 ± 0.08 (Table 2). The post-fallout-expended leaf sample has ^134^Cs and ^137^Cs concentrations of 1.29 ± 0.03 and 1.32 ± 0.04 kBq kg^−1^, respectively, corresponding to a ^134^Cs/^137^Cs activity ratio of 0.98 ± 0.04. Radiocesium activities in the pre-fallout-expended leaves are >2 times higher than those in the post-fallout-expended leaves. The ^137^Cs concentration in our pre-fallout-expended leaf sample is comparable with those in living and fallen leaf samples collected in Iwaki on 18th and 29th March 2011 [23, 24]. Kanasashi et al. [9] made comprehensive measurements of ^137^Cs in Japanese cedar leaves from pre-2010, 2011 and 2012. Among these, ^137^Cs analyses in the Iwaki area produced activities of 0.3–4.6 kBq kg^−1^ in leaves that formed initially in pre-2010, 0.3–2.7 kBq kg^−1^ in those formed initially in 2011 and 0.1–1.5 kBq kg^−1^ in 2012. Clearly, the ^137^Cs concentration in our pre-fallout-expended leaf sample is comparable with their pre-2010 samples, while our post-fallout-expended leaf sample is similar to their 2012 samples.

The ^134^Cs/^137^Cs activity ratio is known to be a reliable source identifier. The average ^134^Cs/^137^Cs ratio at the time of the Fukushima accident (11th March 2011) was found to be approximately 1 [25]. Thus, the identical ^134^Cs/^137^Cs activity ratios in this study are consistent with the initial value observed in worldwide environmental samples, indicating a total dominance of Fukushima accident releases of radiocesium in the samples.

When leaching experiments were conducted on contaminated cedar leaves using deionized water, surfactant and acetone [7], it was found that the reduction in activity for deionized water leaching varied from 0 to 40 %, indicating that up to 40 % of the radiocesium was loosely held on the leaf surfaces. The surfactant and acetone leaching only resulted in further reductions of <10 %. These experiments demonstrated that the remaining radiocesium in the contaminated leaves (both fresh and fallen) was strongly fixed in the leaf tissues, unless the leaf tissues were decomposed. Thus, it can be concluded that radiocesium in the pre-fallout-extended leaves had originated from the direct deposition of Fukushima-derived components. This can be supported by similar ^137^Cs activities in leaves between this study and those collected in a similar region during the early stage of the accident [23, 24].

The radiocesium activity in 20 woody plant species, including Japanese cedar from Abiko, ~200 km southwest of the FDNPP, was investigated in August 2011 [4]. Among the evergreen coniferous species, the average activity in pre-fallout-expended leaves was 2.4 times higher than that in the post-fallout-expended leaves. Furthermore, a distinct variation in the activity among the evergreen coniferous species could be observed for the post-fallout-expanded leaves but not for the pre-fallout-expanded leaves [4]. Kanasashi et al. [9] reported similar ^137^Cs concentrations in cedar pollen and flowers from 2012 and concluded that this was due to uptake and translocation of a significant proportion of the intercepted activity. The average ^137^Cs concentration in leaves laid down from 2012 is ~30 % lower than those laid down prior to the accident but still on the tree. Hence, in comparison with these results, the similar trend observed in this study suggests that the radiocesium in the post-fallout-extended leaves was most likely elevated by the translocation of Fukushima-derived radiocesium from other parts of plant.

However, direct post-accident atmospheric fallout of radiocesium onto the post-fallout-expended leaves cannot be ruled out. The long-term ^134^Cs and ^137^Cs activities in aerosol samples within Fukushima City have been monitored [26] and it has been observed that the levels of ^134^Cs and ^137^Cs in 2012–2013 were significantly higher than the background levels prior to the accident. For example, the average ^137^Cs activities are 2 × 10^−3^ Bq m^−3^ in 2011, 5.2 × 10^−4^ Bq m^−3^ in 2012, and 2.7 × 10^−4^ Bq m^−3^ in 2013 [26]. Monitoring was not conducted during the early stages of the accident (between 11th March 2011 and 17th May 2011), therefore, the actual ^137^Cs activity should be significantly higher than 2 × 10^−3^ Bq m^−3^ reported for 2011. As a result, the difference in atmospheric ^137^Cs between 2011 and 2012 would be more than one order of magnitude, significantly larger than that of contemporary leaf samples. Therefore, the contribution of post-accident atmospheric fallout to the post-fallout-expended leaves should be insignificant.

### ^127,129^I concentrations and ^129^I/^127^I atomic ratios

The pre-fallout-expended leaf sample has ^127^I and ^129^I concentrations of 3.1 μg g^−1^ and 3.6 × 10^9^ atom g^−1^, respectively, corresponding to a ^129^I/^127^I atomic ratio of 2.5 × 10^−7^ (Table 2). On the other hand, ^127^I and ^129^I concentrations in the post-fallout-expended leaf sample were measured to be 0.4 μg g^−1^ and 1.6 × 10^9^ atom g^−1^, respectively, resulting in a ^129^I/^127^I atomic ratio of 8.4 × 10^−7^. The large variation in the ^127^I concentrations in the two samples is comparable with the range (0.22–2.0 μg g^−1^) observed in Japanese pine needles [27]. The ^129^I concentrations are significantly higher than that observed in the background area (<7 × 10^7^ atom g^−1^) at Ito, Japan [27]. The resulting ^129^I/^127^I ratios (2–8 × 10^−7^) are 1–2 orders of magnitude higher than local soils before the Fukushima accident [28], and other background areas within Japan [27, 29]. Hence, these results show a clearly enhanced ^129^I level in the Iwaki forest environment after the Fukushima accident.

^129^I and ^127^I are considered to have different sources in the environment of the Fukushima area. There is no doubt that ^129^I in the atmosphere and soils originated from the Fukushima accident releases, whereas ^127^I has multiple sources. The ^127^I in soil is accumulated from atmospheric deposition and weathering of rocks, of which deposition from the atmosphere might be a major contribution to land near the sea. However, the origins of the atmospheric ^127^I might originate from releases from both the land and sea. Because the post-fallout-expended leaves were collected from the same tree as the pre-fallout-expended leaves, the source from the soil is considerable the same. Therefore, the large difference in ^127^I concentration between these two samples would tend to indicate significant variation (averaged over years of lifespan as shown by ^14^C results) in the atmospheric contribution. As the sampling site is only 15 km from the sea, variations in the contribution of ^127^I from the sea, dispersed and deposited on land by onshore winds, would seem the most likely cause.

Like radiocesium, the high ^129^I activity in the pre-fallout-expended leaves might be attributed to direct foliar uptake from the atmosphere during the Fukushima accident. Indeed, the value is comparable with activities observed in the environment in the Iwaki region by other studies. The ^131^I activity in plant samples collected on 18th March 2011 from two sites (44.4 and 63.7 km from FDNPP) have previously been reported as 516 and 349 kBq kg^−1^, respectively [23]. As there is no significant isotopic fractionation of the releases from the FDNPP, including ^134^Cs/^137^Cs, ^133^I/^131^I, ^129^I/^131^I [25, 30], the ^129^I activity can be calculated from the Fukushima-derived ^129^I/^131^I ratio and the measured ^131^I concentration. Taking a ^129^I/^133^I atomic ratio of 16 [16, 30, 31], the corresponding ^129^I activities can be estimated to be 8.3 × 10^9^ atom g^−1^ and 5.6 × 10^9^ atom g^−1^, respectively. Similarly, relatively high ^131^I activities of 781 and 2603 kBq kg^−1^ have been found in fresh and fallen leaves collected on 29th March 2011 [24]. Using the ^129^I/^133^I ratio of 16 discussed above, we calculate corresponding ^129^I activities of 1.2 × 10^10^ and 4.2 × 10^10^ atom g^−1^, respectively. The ^129^I activity observed in the pre-fallout-expended leaves in this work does not largely differ from the reported values if the different species of plant is taken into account. Therefore, like radiocesium, the Fukushima-derived ^129^I has been deposited on the surfaces of plant leaves and absorbed into the plant tissue through the stomata.

Investigations of iodine in the soil–plant system have indicated that plants can take up iodine from soils [32]. However, the soil-to-plant concentration factor for iodine has been shown to be low due to the strong sorption of iodine by soil components such as organic matter and iron and aluminum oxides, resulting in little translocation from the roots to the aerial parts of the plant. In addition, little iodine in soils has been found to be easily leachable and no correlation between soil and plant iodine has been demonstrated [33]. Therefore, uptake of ^129^I from the contaminated soils is unlikely to be a major pathway to elevate ^129^I in the post-fallout-expended leaves, as the Fukushima-derived ^129^I most likely remained at a relatively high activity inside the tree.

The Fukushima accident caused a significant increase in the ^129^I concentration in the atmosphere, which has been followed by an exponential decline [16]. The ^129^I concentration in 2012 was found to be about 2 orders of magnitude lower than those measured in the early stages of the accident. A similar pattern is also found for the atmospheric activities of ^134,137^Cs in Fukushima City [26]. Therefore, if the ^129^I in the post-fallout-expended leaves was primarily from the ambient atmosphere during the growth period of the leaves, the activity in the post-fallout-expended leaves would be expected to be lower than that in pre-fallout-expended leaves by similar orders of magnitude as observed in precipitation. Clearly, it is not the case in Iwaki cedar leaf samples in which the ^129^I activity in the post-fallout-expended leaves differed from the pre-fallout-expended leaves by a factor of two.

Thus, similar to radiocesium, the lower ^129^I activity in the post-fallout-expended leaves compared to that in the pre-fallout expended leaves is most likely attributed to internal translocation of ^129^I. However, because there are no available ^129^I data measured on the plant a more detailed assessment will be required to confirm this.

## ^129^I/^137^Cs activity ratio and fractionation between radiocesium and radioiodine

The calculated ^129^I/^137^Cs activity ratios are 1.57 × 10^−6^ and 1.66 × 10^−6^ in the pre- and post-fallout-expended leaves, respectively (Table 2). The difference in the activity ratio is small for leaves with different growing periods and consistent with those observed in surface soil from a location of a similar distance to the FDNPP as the leaf samples. For instance, the surface soil samples collected soon after the accident and within a 60 km radius of the FDNPP show ^129^I/^137^Cs activity ratios ranging within 0.3–3 × 10^−6^ [34, 35]. The ^131^I/^137^Cs activity ratios in soil and plant samples collected on 18th March 2011 from two sites in Iwaki have been reported [23]. In the first site (44.4 km from FDNPP), the ^131^I/^137^Cs ratios in soil and plant were 75 and 99, respectively. In the second site (63.7 km from FDNPP), the ^131^I/^137^Cs ratios in soil and plant were 69 and 61, respectively. The corresponding ^129^I/^137^Cs activity ratios are 1.55 × 10^−6^ and 1.68 × 10^−6^, respectively, for soil samples, and 1.36 × 10^−6^ and 2.22 × 10^−6^, respectively, for plant samples (Table 2), determined by taking a ^129^I/^133^I atomic ratio of 16 [16, 30, 31]. Therefore, although there are limited numbers of analyses, the similar ^129^I/^137^Cs activity ratios observed in the post-fallout-expended and pre-fallout-expended leaves implies that the internal translocation might not have caused significant fractionation between radiocesium and radioiodine. However, the observed ^129^I/^137^Cs activity ratios are nearly four times higher than the initial value of the Fukushima-derived component (~4 × 10^−7^) [30, 36]. This suggests that the elemental fractionation between radiocesium and radioiodine mainly occurred during the transportation of the radioactive plumes, because radiocesium would mainly be in the particle-associated form whereas radioiodine can be in both the aerosol and gaseous form. Even within the aerosol, the attached iodine might become gaseous form. On the other hand, some gaseous radioiodine might become attached to aerosol particles at a later stage.

## Conclusions

We report ^12,13,14^C, ^134,137^Cs and ^127,129^I concentrations in pre-fallout-expended and post-fallout-expended Japanese cedar tree leaves collected in Iwaki, Japan. ^14^C concentrations in the leaves reflect the ambient atmospheric ^14^C levels during the year the leaves were sampled or defoliated as well as year(s) prior to this. Higher ^129^I and ^134,137^Cs concentrations in the pre-fallout-expended leaves suggest direct contamination following the Fukushima accident, whereas the relatively low activities in the post-fallout-expended leaves was most likely caused by translocation from other parts within the tree. In comparison with the initial Fukushima-derived ^129^I/^137^Cs ratio, the observed high ratios suggest significant fractionation between radiocesium and radioiodine during processes such as transportation and deposition of radioactive plumes. However, the process of translocation within the tree seem to show insignificant fractionation between radiocesium and radioiodine.
